# Minimally processed fruits as vehicles for foodborne pathogens

**DOI:** 10.3934/microbiol.2023001

**Published:** 2023-01-13

**Authors:** Jessie Melo, Célia Quintas

**Affiliations:** Universidade do Algarve, Instituto Superior de Engenharia, Campus da Penha 8005-139, Faro Portugal and MED, Mediterranean Institute for Agriculture, Environment and Development, Universidade do Algarve, *Campus* de Gambelas, 8005-139 Faro, Portugal

**Keywords:** fresh-cut fruit, foodborne pathogens, cross contamination, microbiological quality, control measures

## Abstract

The consumption of minimally processed fruit (MPF) has increased over the last decade due to a novel trend in the food market along with the raising consumers demand for fresh, organic, convenient foods and the search for healthier lifestyles. Although represented by one of the most expanded sectors in recent years, the microbiological safety of MPF and its role as an emergent foodborne vehicle has caused great concern to the food industry and public health authorities. Such food products may expose consumers to a risk of foodborne infection as they are not subjected to prior microbial lethal methods to ensure the removal or destruction of pathogens before consumption. A considerable number of foodborne disease cases linked to MPF have been reported and pathogenic strains of *Salmonella enterica*, *Escherichia coli*, *Listeria monocytogenes*, as well as Norovirus accounted for the majority of cases. Microbial spoilage is also an issue of concern as it may result in huge economic losses among the various stakeholders involved in the manufacturing and commercialization of MPF. Contamination can take place at any step of production/manufacturing and identifying the nature and sources of microbial growth in the farm-to-fork chain is crucial to ensure appropriate handling practices for producers, retailers, and consumers. This review aims to summarize information about the microbiological hazards associated with the consumption of MPF and also highlight the importance of establishing effective control measures and developing coordinated strategies in order to enhance their safety.

## 1. Introduction

Fruits comprise a large group of plant foods that represent an important source of essential nutrients for a balanced diet. They also provide bioactive phytochemicals, such as flavonoids and phenolic compounds, associated with several health-promoting benefits [1]. In recent years the European Union (EU) health institutions have run campaigns recommending the daily consumption of at least “5 a day” fruit and vegetable portions. In addition, the World Health Organization (WHO) recommends a minimum of 400 g per capita [2]. These campaigns have been strongly supported by the increasing evidence of an enriched fruit diet associated with a lower risk of cardiovascular diseases and several types of cancer [3]. In fact, a daily fruit intake seems to have a positive impact on the prevention of a great number of chronic diseases [4].

Worldwide significant changes in lifestyles and major shifts in consumer trends have taken place. Such changes reflect the demand for a new and wider range of fresh products, which along with a shorter available time for home cooking, led the food industry to an emerging market of ready-to-eat fresh products. As a response to a growing demand for convenient, healthy, and easy-to-prepare fresh products, a wide range of minimally processed fruit (MPF) has been developed [5]. These products seem to represent a good alternative to today's lifestyle as they provide a safe handling and a rich source of nutrients, along with an attractive presentation [6]. They can also allow the consumer to reduce waste, since only the edible part of the product is taken home.

This mini-review aims to gather information about the microbiological and safety issues associated with the consumption of processed fruit taking into consideration their highly perishable nature, as well as provide some insight into prevention measures.

## 2. Processing of fruits

MPF are products that have undergone physical changes but retain the freshness and the natural properties of the original fruit. Also called as “ready-to-eat”, “pre-cut” and “fresh-cut”, these fruits are submitted to unit operations, which include selection, cleaning, washing, trimming, peeling, cutting/shredding/mashing, sanitizing, and finally packing. During production they are not submitted to microbial lethal techniques (e.g., pasteurization) that might reduce microbiological risks, so these foods can be potential vehicles for the transmission of pathogenic bacteria, viruses, toxins or spore-forming microorganisms [7]. As a result, fresh-cut fruit must be stored, distributed and marketed under refrigeration to achieve a satisfactory shelf-life, which can last between 7 to 20 days if an adequate cooling temperature is maintained. However, the tendency to extend the shelf-life of refrigerated foods may facilitate the proliferation of psychrotrophic microbial contaminants, pathogenic or spoilers. Concern about the microbiological safety of MPF has increased due to the emergence of foodborne infections connected to their consumption and to the increase of vulnerable populations (seniors, weakened immune systems individuals). A number of disease cases have been reported in the United States of America (USA) and EU countries representing a public health hazard and a negative impact on this food industry sector. Contamination can occur at any step of production, processing, distribution, and also as a result of consumer practices. Therefore, understanding the key factors in the transmission chain will be a valuable contribution to establishing best practices and prevention measures. After harvesting the fruits are processed through a series of operations, summarized in Table 1.

**Table 1.** Main steps/operations of industrial processing of minimally processed fruit (MPF) [7],[88]–[90].

Selecting	○ Selection of good quality fruits with adequate color, acidity, Brix ○ Advanced maturation stages limit the shelf-life of MPF ○ Early maturation stages impact negatively the sensorial characteristics of MPF
Washing	○ Removal of field residues (dust, pesticides, insects, etc.) ○ Cooling immediately after washing to lower internal temperature and delay the biological processes that accelerate the maturation
Peeling	○ Removal of the fruits outer layer when it is not edible or when the final presentation requires it ○ Manual, mechanical, enzymatic. Hot water or high-pressure steam may be used ○ All peeling should be done in the least abrasive way to prevent the invasion of internal tissues by microorganisms (internalization) and avoid darkening
Cutting	○ Dimension reduction operations: slicing, chopping, grating, cutting into cubes or into sections ○ Manual or mechanical ○ Good sharp cutting tools should be used to reduce physical damage to the cells
Washing (2^nd^) and disinfection	○ Done by spraying with water or immersing the fruit for a pre-established period of time in chilled water in tanks (1–10 °C) containing an adequate concentration of disinfectants ○ Most common disinfectant: chlorine (50–200 ppm) ○ Step that reduces the microbial load during MPF processing ○ Microbiological and chemical quality of the water should be regularly monitored to avoid cross-contaminations
Rinsing	○ Removal of excess surface water and disinfectants residues from MPF ○ Residual surface drops of moisture and surface exudation of freshly cut fruits may stimulate the growth of fungi and bacteria ○ Avoid damage to the fruit tissues
Packaging	○ MPF weighing ○ MPF packaging ○ Metal control ○ Bags, boxes or trays and different types of protective films ○ Refrigerating temperatures

## 3. Microbial hazards

**Figure 1.**
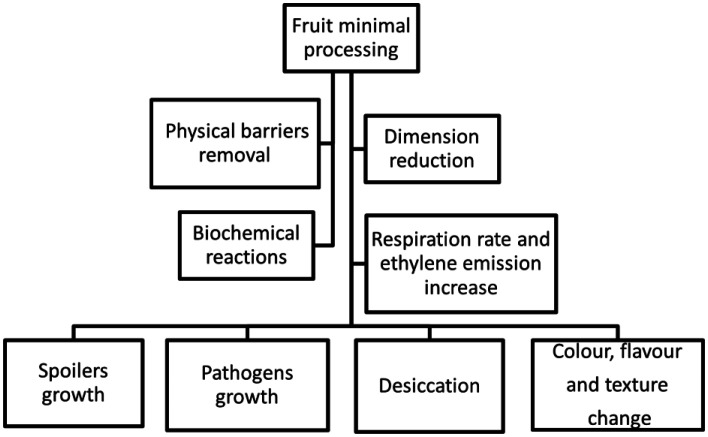
Impact of minimally processing on the quality of the final product.

MPF are raw foods ready to consume, characterized by cut, non-sterilized, physiologically active surfaces rich in nutrients and water, and not thermal or chemically preserved. The minimal processing (peeling and dimension reducing) contributes to the increase of the tissue respiration rate as well as other biochemical reactions, such as the production of ethylene in the cells, which generates heat and accelerates degradation. As a result, in addition to spoilers and pathogens growth, these products are also susceptible to several visual changes during shelf-life (color, flavor, texture, and surface desiccation) (Figure 1).

Due to the survival and growth of pathogenic microorganisms on MPF, their consumption has been connected with infections caused by bacteria, viruses and parasites [8],[9]. Nowadays, consumers have a great choice of fruits in the retail markets, which are available in several forms: fresh, minimally processed, canned, frozen, or dried. Processing of these kinds of fruit products consists of a series of operations where contamination and cross-contamination can take place at both the industry or home level. Therefore, as previously mentioned, these commodities are not submitted to any surface pasteurization or cooking, which make them a significant route for foodborne pathogens representing a threat to consumers' health. The major pathogenic microorganisms associated with the consumption of fruit are *Salmonella enterica*, *Escherichia coli*, *Listeria monocytogenes*, as well as the Hepatitis A and Norovirus viruses [7],[10],[11].

### 3.1. Salmonella enterica

*Salmonella enterica* is one of the leading agents of foodborne illnesses and is responsible for thousands of deaths every year. Infections caused by this pathogen are a major concern to public health and the food industry worldwide, and both animal and non-animal food sources are potential vehicles of disease transmission. In the last two decades a great number of *S. enterica* outbreaks have been traced to fresh, fresh-cut and frozen fruits consumption, which were associated to a high diversity of serovars. A very high number of serotypes/serovars of *S. enterica* are distinguished on the basis of somatic, flagellin and capsular antigens, resulting in over 2,500 antigen combinations [12].

Depending on the host and serotype, *S. enterica* is the etiologic agent of enteric fever (typhoid fever), enterocolitis/diarrhea and bacteremia. Serotypes *S*. Typhi and *S*. Paratyphi are particularly adapted to humans, causing typhoid fever. Non-typhoid *Salmonella* serotypes are the most frequent salmonellosis agents in developed countries. Several studies have demonstrated the ability of *Salmonella* spp. to multiply on the leaf surface of young plants suggesting that plants may constitute alternative hosts for *Salmonella* and play an important role in their transmission to animals [13]–[15].

The common reservoir of *S. enterica* is the human, domestic and wild animal gastrointestinal tract. *S. enterica* enters the soil and agricultural environments through animal feces and can directly contaminate plants and surface waters used for irrigation and pesticide/fertilizer preparation.

Salmonellosis occurs after ingestion of contaminated food or water or as a result of contact with symptomatic or asymptomatic carriers. Bacteria survive the acidity of the stomach and colonize the intestine where they invade the epithelial cells and are trapped in vacuoles. Vacuoles containing the bacteria can be destroyed, releasing the microorganisms into the cytosol of host cells and replication can occur allowing *Salmonella* to display an intracellular lifestyle [16],[17].

According to the microbiological criteria of the EU [18] *Salmonella* must be absent in various food categories including vegetables and MPF. If there is fecal food contamination the transmission of the microorganisms will occur, particularly under growth promoting conditions when food is stored at inadequate temperatures. Numerous studies have demonstrated the survival and growth of *Salmonella* spp. in fruit. Strawn and Danyluk [19] observed an increase in *Salmonella* cells on cut mangos and papayas, stored at 12 °C, and survival for 28 days when storage was at 4 °C. They also reported the survival of *Salmonella* on cut mangos and papayas after 180 days of freezing. An increase in *Salmonella* populations in fresh-cut peaches stored at 20 and 25 °C was also addressed by Alegre et al. [20]. Ukuku et al. [21] observed the survival of *Salmonella* population in sliced Cantaloupe melon stored at 5 °C for 7 days and an increase in its growth rate in samples stored at 10, 15 and 20 °C. Palekar et al. [22] reported the survival of *Salmonella* Poona for 21 days in Cantaloupe melon slices stored at 5 °C. In ‘Rocha’ pear, *Salmonella* grew at 12 and 20 °C, reaching a population of more than 8 log cfu/g, in a period of 24 hours. However, in three days at 8 °C, it only increased about 1 log cfu/g and at 4 °C it did not multiply [23].

### 3.2. *Escherichia coli* O157:H7

The pathogenic strain of *Escherichia coli*, namely *E. coli* O157:H7, is a relevant pathogen related to MPF safety since it has been implicated in some outbreaks and sporadic cases. *E. coli* strains are members of the gastrointestinal microbiota of man and mammals. Fecal matter can contaminate food and water, including irrigation and recreational waters. Human infections by pathogenic *E. coli* strains occur after the consumption of contaminated food such as undercooked meat, contaminated fresh vegetable and fruit or through contact with contaminated animals [24]. Disease transmission through person-to-person contact may also occur when proper hygiene care is not ensured. Fruit, like any other food of plant origin, can be contaminated by cross-contamination through contact with raw materials and meat. Food handlers (symptomatic or asymptomatic carriers) may also be responsible for the transmission of the bacteria [25],[26].

The ability of *E. coli* strains to survive and grow in environments other than the gastrointestinal tract represents a public health threat. *E. coli* has been isolated from environments such as soil, manure and irrigation water. However, it has also been found in the internal tissues of lettuce [27] and plant roots [28]. MPF permit *E. coli* dissemination in food industry processing and packaging environments, contributing to the transmission through the food chain when good manufacturing practices are not complied. *E. coli* O157:H7 can survive and multiply outside the gut and has been implicated in various disease outbreaks related to the intake of fresh-cut fruits and vegetables [7]. Abadias et al. [29] found the survival of the *E. coli* O157:H7 population inoculated in fresh-cut pineapple and melon, packed in a modified atmosphere, and stored at 5 °C for 15 days. In peaches an increase of 2 log cfu/g was observed in the *E. coli* O157:H7 population at 20 and 25 °C [20]. Strawn and Danyluk [19] reported that *E. coli* O157:H7 was able to grow in fresh-cut mango and papaya stored at 23 °C, surviving for 28 days when samples were stored at 4 °C and 180 days when frozen at -20 °C. In ‘Rocha’ pear *E. coli* showed significant growth at 12 and 20 °C, reaching high populations (>8 log cfu/g, in 24 h). At 8 °C the microorganism increased more than 1 log cfu/g in 3 days, although at 4 °C no proliferation occurred [23].

### 3.3. Listeria monocytogenes

*Listeria monocytogenes* is a robust pathogen with relevance to public health and the food industry as it can lead to listeriosis, a severe foodborne disease affecting mostly risk groups, such as children, pregnant women, elderly and immunocompromised people. Although relatively rare compared to other foodborne infections, listeriosis is associated with a high mortality rate and clinical cases can lead to 20–30% deaths [30]. This bacterium is widely found in the environment (soil, water, manure, decaying vegetation), can persist in mammalian and avian feces, and also in multiple food processing environments. Its ability to survive and grow in multiple niches is supported by a complex system of tolerance responses [31]. Those responses help the pathogen to survive to several inhibitors frequently found in the food industry, such as disinfectants, sanitizers, low-pH conditions, osmotic pressure and eventually allowing it to remain in this environment for long periods [32]. Strains that are involved in foodborne diseases seem to be also linked to biofilm formation, which makes them even more difficult to eliminate from industrial food facilities once they are established [33]. As a psychrotrophic, *L. monocytogenes* can survive and multiply at low temperatures and may reach dangerous levels in foods kept under refrigeration. The ability of *L. monocytogenes* to grow under temperature abuse conditions (3 days at 4 °C followed by 5 days at 8 °C) was also reported on fresh-cut ‘Conference’ pears packed under modified atmosphere during their shelf-life [34]. Furthermore, this tolerance response system permits the pathogen to have a successful transition from food to the gastrointestinal host system, leading to cell invasion and the establishment of infection [35].

This pathogen has been associated with a great variety of foods including dairy, poultry, meat, seafood, fruits, and vegetables [36]. In the last decade, the importance of the consumption of non-animal foods, as a cause of listeriosis has been increasingly recognized in fresh, fresh-cut, and frozen fruits [37],[38]. Along with *S. enterica* and *E. coli* O157:H7, *L. monocytogenes* became a microorganism of great concern to fruit and vegetable safety [39]. In 2011, the consumption of contaminated cantaloupe was responsible for a multistate outbreak in the USA resulting in 147 people infected and 33 deaths. In 2014, another multistate listeriosis outbreak associated with the ingestion of caramelized apples cross-contaminated at the packing facility caused 35 hospitalizations and three deaths [40]. Environmental samples collected at the apple packinghouse and clinical isolates revealed the presence of similar strains [41]. In the same year, an outbreak linked to stone fruits (peaches, plums and nectarines) contaminated with *L. monocytogenes* was also reported [42].

For a long-term period, it had been generally assumed that intact fruits were only contaminated by microorganisms at the external surface and the internal acidic environment of most fruits would prevent bacterial contamination. However, the whole fruit can also serve as a vehicle for foodborne disease transmission. Caramel apples were assumed to be a minimal risk food for listeriosis due to the internal fruit acidity and to the low water activity of caramel, but growth of *Listeria* had already been reported in fresh apples by Conway [43]. The survival, growth, and internalization of *L. monocytogenes* in fresh and processed fruits have been well documented and they are now recognized as one of the emergent food products at risk of listeriosis transmission [42]. Graça et al. [23] studied the growth of *Listeria* spp. on fresh-cut ‘Rocha’ pear and reported the bacteria capacity to multiply at 20, 12, 8, and 4 °C, despite needing adaptation periods inferior to 24 h at 8 and 4 °C. According to Zeller et al. [44] the growth of *L. monocytogenes* on MPF correlated significantly with the pH. None of the tested MPF with a pH below 4 showed a significant proliferation of this bacterium.

*L. monocytogenes* is usually killed by cooking and high-temperature methods, so food products eaten raw, such as fresh, fresh-cut and frozen fruits, are at the highest risk. According to the EU regulation, the absence of *L. monocytogenes* is not required in all ready-to-eat products. In food products that support growth, the levels of *L. monocytogenes* cannot increase higher than 100 cfu/g over the shelf-life [18]. However, in the USA there is zero tolerance for this pathogen.

Taking into consideration the characteristics of *L. monocytogenes*, it becomes a difficult, if not impossible task, to completely eliminate it from processing environments. Furthermore, the last reported outbreaks, sporadic cases and several recalls of fresh and fresh-cut fruit due to the presence of *L. monocytogenes* have highlighted the serious hazard of this food products safety and the urgent need for improving control measures in the fruit supply chain [45].

### 3.4. Norovirus

Food-borne viral infections have been increasingly recognized as one of the major causes of human diseases. Norovirus (NoV) has emerged worldwide as a leading agent of acute gastroenteritis outbreaks, causing million cases annually. Although characterized as mild infections, in risk groups, such as children under five, elderly or immunocompromised people, these diseases can be responsible for severe outcomes with a high rate of hospitalizations and deaths [46]. NoV are enteric pathogens, non-enveloped single-strand positive-sense RNA viruses, classified into the *Caliciviridae* family, which comprises a genetically and antigenically diverse group, having at least seven known genotypes (GI-GVII). Three genotypes (GI, GII and GIV) can cause disease in humans, being the GII the most frequently reported [47].

NoV and hepatitis A virus (HAV) are the main foodborne viruses associated with the consumption of fresh, MPF, and frozen berries [2], as well as the pathogenic bacteria mentioned before. Fresh and frozen fruits, such as grapes, raspberries, blueberries and strawberries have been reported as vehicles of an increasing number of NoV outbreaks [48]–[50]. One of the most severe NoV outbreaks occurred in Germany in 2012, when 11,000 people were affected by the consumption of frozen berries imported from China [51]. In March 2009 a massive outbreak associated with frozen raspberry ingestion caused 500 cases in a primary school in Finland [52]. Fruits pose a significant risk to foodborne viral transmission as they are consumed raw and are not submitted to lethal treatments. Contamination can occur at any step of the production chain. Even an endophyte contamination of fruit has been suggested [53]. Water is a critical vehicle in the virus's infectious cycle. Non-enveloped RNA viruses are usually present in human sewage in high loads. They are neither completely removed or inactivated by conventional sewage treatment processes and can be discharged into the environment. The use of contaminated water in agriculture or in processing operations (washing steps) are major vehicles of viral transmission. The shedding of virus particles by symptomatic or asymptomatic carriers can spread the disease through handlers and surfaces/equipment contributing to the viral transmission [54]. Although the majority of noroviruses outbreaks were caused by frozen berries the consumption of minimally processed fruits can also represent a potential risk for the transmission of this foodborne disease etiological agent [55]–[57].

NoV shows high resistance to environmental stressors, such as heat, high/low pH, drying, light and UV exposure and also to chemical and physical disinfection treatments [58]. This persistence allows them to remain infective in foods for periods from 2 days to 4 weeks. As most foodborne viruses they are supposed to have very low infectious doses and fewer than 10 viral particles are required to cause disease [59]. NoV infections usually have a 12–48 h incubation period followed by symptoms, such as vomiting, diarrhea, abdominal cramps, and low-grade fever.

NoV has also shown to be resistant to the most common food preservation methods and can survive chilling, freezing, acidification, reduced water activity and modified atmosphere packaging [60] Minimally processing can induce cross-contamination of uncontaminated fruit during all post-harvest operations/utensils, in addition to the involved workers' health condition [61]. The long-term persistence of NoV on surfaces of food preparation plays a significant role in viruses dissemination [62]–[64].

Screening food products for the presence of foodborne viruses is challenging due to the physical and chemical properties of the food matrices. On the other hand, cultivating foodborne viruses in cell cultures is yet not possible. Therefore, the detection methods of these microorganisms rely upon the molecular techniques that are crucial in the investigation and prevention of outbreaks. The last decade has shown significant development and optimization of new and sensitive methods of NoV detection and the RT-PCR assay has become the gold standard for food products. Rapid laboratory diagnosis can be an important tool in controlling NoV outbreaks and guiding the choice of prevention measures such as cleaning/disinfection protocols, isolation of infected food handlers and surveillance of the pathogen in the food processing environment.

## 4. Sources of contamination

MPF are susceptible to microbiological contamination at any step of the production chain and eventually in the consumers' kitchen. The contamination can originate from human, animal or environmental sources and take place during the pre-harvesting, harvesting, and post-harvesting stages.

### 4.1. Pre-harvest

One of the pre-harvesting sources of contamination is the soil where plants are cultivated since soil is a natural habitat of various microorganisms and crop production is often fertilized by untreated manure/human biosolids, which can harbor high levels of pathogenic microorganisms [65]. Low quality water sources used for irrigation, such as reutilized or waste waters, can as well contribute to significant contamination. Meteorological conditions also matter and the occurrence of rain or drought, the presence of dust, aerosol, and feces may have a great influence on microbiota proliferation [66].

### 4.2. Post-harvest

During the post-harvest all the steps are susceptible to cross-contamination and can eventually lead to an increase of the microbial population on the final products. Fruit washing may not remove microorganisms as they can remain attached to plant surfaces or become internalized in the edible parts of the fruit [67]. The efficacy of antimicrobials used in the disinfection never reaches 100% as their active principles may be consumed in chemical reactions with organic matter or microorganisms may be protected in biofilms or internalized in the fruit tissues. The methods used to decontaminate the fruits are based on physical or chemical processes or a combination of both. The most widely used disinfecting agent in the fresh food processing industry is chlorine. However, the levels of bacterial reductions obtained when using chlorine solutions in MPF, at the concentrations (50–200 ppm) and contact times allowed (1–2 minutes), are around 1 to 2 log cfu/g [68]. The most active form of chlorine is hypochlorous acid whose highest activity occurred between pH values of 5.0 and 6.0 as measured by Marin et al. [69]. In addition, the same authors showed that the most adequate pH regulators were the non-organic ones, such as phosphoric acid when compared to the organic pH regulators (for example citric acid). The microbiological characteristics of the water used in the washing procedures are crucial to avoid cross contaminations. For example, Penteado et al. [70] observed that mangoes (‘Tommy Atkins' variety) can get contaminated with *S. enterica* when contaminated water at 47 °C was used in the washing.

**Figure 2.**
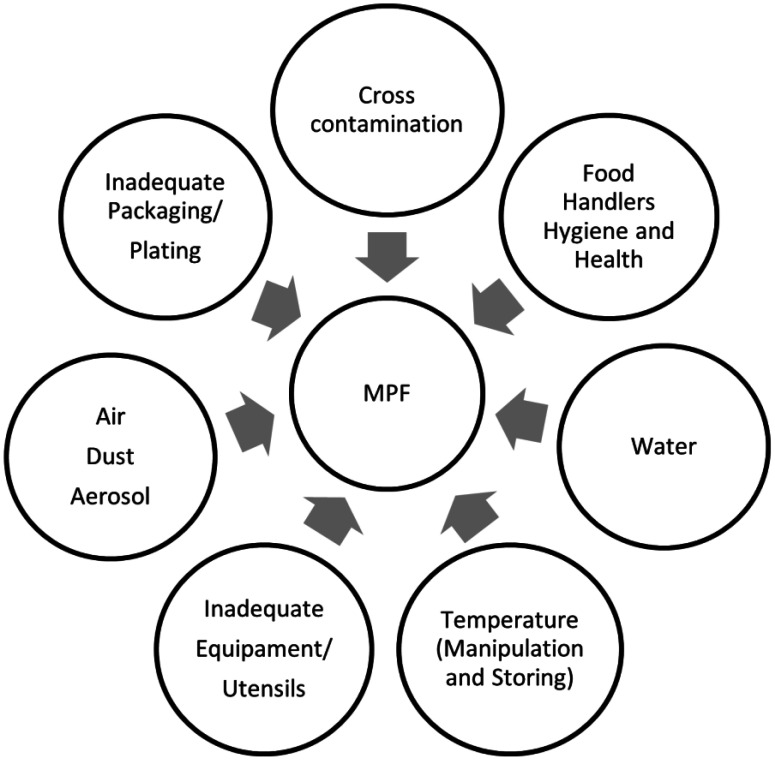
Main sources of MPF microbial contamination at the post-harvest level.

The post-harvest microbial contamination of MPF is also associated with a great number of sources: the use of contaminated containers/utensils, unhygienic conditions of food handling, unhygienic surfaces/equipment, packaging material, transport vehicles, and inadequate storing temperature. For example, the peeling and size reduction operations as well as food contact surfaces also present the risk of contamination and recontamination due to the contact of contaminated equipment with fruits (Figure 2).

A study conducted in industrial plants revealed high levels of total aerobic microorganisms, higher than 20 cfu/cm^2^, on all food contact surfaces, namely in peeling equipment, knives, and cutting boards, among others [71]. The same study reported high counts of the Enterobacteriaceae family (β-Glucuronidase positive *E. coli*) on cutters (90 cfu/cm^2^) and the chopping boards (76 cfu/cm^2^). In fact, once introduced, some microorganisms are able to persist and form biofilms if the design, composition and topography of surfaces allow. Poor design characteristics, such as horizontal surfaces, right angles, welds/joins, and container/tank corners are critical regarding hygiene, being more difficult to clean and, more susceptible to the development of biofilms, as studied in fresh-cut washing containers [72]. Another example of the importance of well-designed equipment was the one related to the listeriosis outbreak caused by cantaloupe consumption, in 2011, which indicated a postharvest contamination of fresh melons. Changes in the equipment used for the washing and drying process of these fruit products created favorable environmental conditions for microbial growth. This equipment, not designed for an efficient sanitization, allowed the contact of its components (such as brushes and felt rollers) with the fruits enabling pathogen adhesion and colonization, which may have spread to other food contact surfaces and promoted microbial contamination [73].

Packaging is a critical step in MPF processing and should be carried out with maximum hygiene. The modified atmosphere packaging (MAP) is one of the available techniques at this level to reduce the microbial load and extend the shelf-life of fresh fruit and MPF. This method consists of the modification of the internal atmosphere of a package, replacing the oxygen content with carbon dioxide or nitrogen. The process decreases the respiration rate, ethylene production, and enzymatic browning, providing a delay of ripening and suppressing the growth of indigenous aerobic microbiota, then maintaining the appearance of the fruit. Atmospheres with low O_2_ increase the anaerobic metabolism of fruits and fermentation which may produce acetaldehyde and taste-altering compounds. Concerning microbial growth, atmospheres with low O_2_ concentrations inhibit the growth of most aerobic degrading microorganisms, such as Gram-negative bacteria or filamentous fungi. However, under certain conditions, the growth of spoilage facultative anaerobic yeasts and anaerobic or microaerophilic psychrotrophic pathogenic microorganisms, such as *Clostridium* spp. and *L. monocytogenes* may be stimulated. The growth and toxin production of *Clostridium botulinum* is of particular concern. On the other hand, the extension of shelf-life may increase the available time and the possibility of pathogens, if present in these food products, to grow [7].

The use of MAP for fresh-cut fruits requires careful selection of the film and package type for each product. Temperature control at storage and distribution is also an important factor for an effective MAP system. However, the effect of MAP on microorganisms can vary depending mainly on the storage conditions and the type of product. According to Corbo et al. [74], MAP containing 5% O_2_ and 30% CO_2_ had no effect on *L. monocytogenes* growth on cactus-pear; the pathogen survived and also grew at refrigeration temperatures (4 and 8 °C). Furthermore, Abadias et al. [29] observed that high levels of CO_2_ (11, 25, 39 %) under 1, 2 and 3 days of storage had little or no inhibitory effect on *E. coli* growth on fresh-cut melon kept at 25 °C. On the other hand, *Salmonella* spp. showed high variability in response to MAP conditions in different studies. Raybaudi-Massilia et al. [75] reported a slight decrease in *Salmonella* Enteriditis population on fresh-cut apples and pears at 5 °C, under MAP conditions. On the contrary, an increase in *Salmonella* Michigan population on fresh-cut peaches was registered when samples were stored at 25 °C with a CO_2_ level higher than 20% [20].

However, it is important to highlight that some of the mentioned hazards are predictable but many of them may be unexpected, particularly in cases of endophytes, when microorganisms produce biofilms, or internalize in the fruit tissues.

## 5. Internalization of pathogens

The ability of human pathogens to internalize plant tissues has been addressed in a great number of studies. Human pathogens can survive the harsh soil environment, adhere to, and actively invade plants. They can enter plant tissues either through natural elements (stomata, roots junctions, flowers), as well as through damaged tissues (cut surfaces, wounds) [76],[77]. The contact with pathogens leads to infiltration and colonization of the plant tissues and this process can take place both at pre-harvesting and post-harvesting phases.

The presence of human enteric pathogens in crop fields can be a result of contaminated irrigation water, climate conditions (rain and wind), insect and nematode vectors [78]. Soils and plants can be contaminated by contact with raw manure or sewage and the enteric pathogens persistence in this kind of environment has been observed [79]. *E. coli* O157:H7 and *S. enterica* have been isolated from the feces of birds and domestic animals and transmission to soil and plants has been well documented [80]. MPF surfaces are especially favorable to the entrance of pathogens since current sanitizing practices are not effective to remove or inactivate internalized bacteria. Surface bacteria may not be totally removed by washing procedures with hypochlorite at the standard concentrations during industrial processing [81].

Multiple studies have addressed the potential for the systemic transfer of internalized bacterial cells within plant tissues. Holden et al. [82] were able to show that internalized *E. coli* O157:H7 and *S. enterica* remained viable and cultivable in the leaves of lettuce, spinach and tomato. Deering et al. [83] also observed the viability and persistence of those pathogens after a period of three weeks on the same vegetables, which is relevant to the harvesting time of those plants. In addition, the pathogenicity and virulence of those bacteria are not affected by the plant colonization [84].

To be able to colonize the surface or interior of a plant human pathogenic bacteria must compete with the naturally present microbiota. To utilize plant nutrients and ensure their persistence in the tissues those microorganisms may depend on the presence of natural endophytes (bacteria or fungal populations), which can provide carbon and energy sources (via degradation of cell wall polymers or induced secretion of sugars) that otherwise would be inaccessible to the pathogens [85],[86]. Successful pathogens probably accept nutrient efflux mechanisms of the host to redirect nutrient flux. However, nutrient acquisition used by bacterial pathogens and the mechanisms they use to alter host physiology, notably the efflux of sugars to support growth, are poorly understood [56]. Future research is needed on the identification of bacterial effectors and their target genes in plant cells that facilitate pathogen nutrition [56],[87]. A better understanding of the dynamic interactions between the plant endophytes and soil microbiome as sources of contamination during plant growth are relevant to the fresh produce production chain. The internalization of pathogenic microorganisms and the occurrence of endophytism in vegetables make microbial contamination increasingly unpredictable and need to be considered in risk assessment.

## 6. Preventive measures during processing

It is crucial to apply preventive measures to reduce foodborne pathogens throughout the farm-to-fork chain of fresh-cut fruit production and ensure safety and quality, decreasing the risk of any potential outbreak due to their consumption (Table 2).

**Table 2.** Preventive measures to reduce microbiota contamination and growth (pathogens and spoilers) during post-harvest stages of MPF fruits (HACCP- Hazard Analysis and Critical Control Point).

Preventive measures	Operations/actions
Use good quality water in:	Cleaning Washing Disinfecting
Disinfection:	Chemical (chlorine, organic acids, electrolyzed water) or physical (irradiation) processes or a combination of both Most common disinfectant: chlorine
Select refrigeration temperatures during: (Avoid temperature abuse)	Storage Transportation Distribution Exhibition/Marketing In restaurants, hotels, at home
Avoid moisture in the processing environment and on fruits surface:	Drying after washing and rinsing (Avoid condensation in packages)
Use proper and sharp cutting equipment:	Reduction of fruit tissue destruction
Segregation is a mandatory rule to decrease cross-contamination:	Segregate processed from unprocessed fruit Segregate animal from plant origin food
Select hygienic-designed equipment and infrastructures:	Choice of containers without corners, welds, right angles
Select cleaning and disinfection plans of food plants, food contact surfaces and equipment with the appropriate frequency:	Elect processing facilities with a hygienic architecture Choose hygiene-designed equipment Disinfectants are not 100% efficient Avoid biofilm production Prevent aerosol formation
Adopt good manufacturing practices:	Prevent fecal contamination Stimulate the adoption of strict personal hygiene Provide education for food handlers Implement HACCP
Be aware that microorganisms can:	Express virulence Evolve and mutate Adapt to disinfectants/biocides Grow during shelf-life Produce biofilms Internalize the tissues of the fruit Possess an endophytic profile

## 7. Conclusions

The safety of MPF is a relevant issue since these food products can act as vehicles for foodborne infections and diseases associated with their consumption are of concern to public health services and to the food industry. Controlling them requires specific guidelines aimed at reducing the risks of contamination, from pre to post-harvest stages until the consumers' table. Implementation of good and hygienic agricultural and manufacturing practices prior, during and after the processing of MPF, including an efficient cold chain, along with compliance to HACCP principles by all the involved stakeholders, are key measures to mitigate outbreak risks and minimize the economic impact of MPF spoilage. Regular monitoring and surveillance from food safety authorities and regulatory agencies are also crucial strategies to increase the quality, safety, and shelf-life of MPF. Nevertheless, epidemiological traceability of these products as human pathogens carriers is difficult to achieve. Further studies are required on microbial internalization, endophytism, and microbiological survival and growth on MPF at different stages of processing. Professionals, from production to commercialization, should be made aware that microorganisms evolve, and adapt to biocides/disinfectants and various stressful conditions, in addition to their ability to internalize, which makes microbial contamination highly unpredictable. Control measures should focus on prevention and be strongly robust to deal with the growing unpredictability of microbial risks.
